# Genetic risk for atrial fibrillation could motivate patient adherence to warfarin therapy: a cost effectiveness analysis

**DOI:** 10.1186/s12872-015-0100-7

**Published:** 2015-09-29

**Authors:** Dov Shiffman, Marco V. Perez, Lance A. Bare, Judy Z. Louie, Andre R. Arellano, James J. Devlin

**Affiliations:** Quest Diagnostics, 1401 Harbor Bay Parkway, Alameda, CA 94502 USA; Stanford University School of Medicine, 291 Campus Drive, Stanford, CA 94305 USA

**Keywords:** 4q25 genetic test, Adherence, Cost effectiveness

## Abstract

**Background:**

Atrial fibrillation (AF) increases risk of stroke, and although this stroke risk can be ameliorated by warfarin therapy, some patients decline to adhere to warfarin therapy. A prospective clinical study could be conducted to determine whether knowledge of genetic risk for AF could increase adherence to warfarin therapy for patients who initially declined therapy. As a prelude to a potential prospective clinical study, we investigated whether the use of genetic information to increase adherence could be cost effective.

**Methods:**

Markov model assessed costs and utilities of two care strategies for AF patients who declined warfarin therapy. In the usual care strategy patients received aspirin. In the test strategy genetic risk for AF was assessed (genotype of the 4q25 locus) and some patients with a positive genetic test (≥1 risk allele) were assumed to adhere to warfarin therapy. The remaining patients received aspirin. The incremental cost-effectiveness ratio (ICER) was the ratio of the costs differential and the quality adjusted life-years (QALYs) differential for the two strategies.

**Results:**

We found that the 4q25 genetic testing strategy, compared with the usual care strategy (aspirin therapy), would be cost-effective (ICER $ 47,148) if 2.1 % or more of the test positive patients were to adhere to warfarin therapy. The test strategy would become a cost saving strategy if 5.3 % or more of the test positive patients were to adhere to warfarin therapy. If 20 % of test positive patients were to adhere to warfarin therapy in a hypothetical cohort of 1000 patients, 7 stroke events would be prevented and 3 extra-cranial major bleeding events would be caused over 5 years, resulting in a cost savings of ~ $250,000 and a net gain of 9 QALYs.

**Discussion:**

A clinical study to assess the impact of patient knowledge of genetic risk of AF on adherence to warfarin therapy would be merited because even a modest increase in patient adherence would make a genetic testing strategy cost-effective.

**Conclusion:**

Providing patients who declined warfarin therapy with information about their genetic risk of AF would be cost effective if this genetic risk information resulted in modest increases in adherence.

## Background

Atrial fibrillation (AF) is a common heart rhythm disorder affecting about 2.4 million people in the US [1, 2], and this number is projected to exceed 5.6 million by 2050 [3]. AF is associated with a 5-fold greater risk of embolic stroke [4, 5] and accounts for 75,000 to 100,000 strokes per year in the US [4]. The risk of stroke due to AF can be reduced by about 50 % with oral anticoagulants such as warfarin [6, 7], and current American Heart Association and American Stroke Association (AHA/ASA) guidelines recommend prophylactic therapy with warfarin for high and moderate risk AF patients [8]. Despite strong evidence supporting its efficacy, adherence to warfarin therapy is low—among patients who started warfarin therapy for AF, more than 1 in 4 patients discontinue warfarin therapy within one year [9, 10]. Low adherence to warfarin therapy is driven by patient concerns about potential bleeding events and the need for continued periodic blood tests (prothrombin time/International Normalized Ratio (INR)) to monitor the patient’s response to warfarin [11–14].

Any strategy that would increase adherence to warfarin therapy among eligible AF patients has the potential to prevent fatal and non-fatal stroke events. Strategies for increasing adherence to warfarin that have been previously investigated include counseling [15, 16], the use of decision aids [16], as well as self-testing and self-management programs [17]. Providing patients with information about their genetic-based risks also has the potential to improve adherence and ultimately clinical outcomes. A recent study reported that patients with a genetic diagnosis of familial hypercholesterolemia were ~50 % more adherent to treatment than were patients without a genetic diagnosis [18]. Similarly, patients’ knowledge of genetic test results increased adherence (63 vs. 45 %) to statin therapy in the AKROBATS study [19].

Since gene variants in the 4q25 region of the human genome are associated with increased risk of AF and stroke [20], providing 4q25 genetic test results to patients might increase adherence to warfarin therapy. If this strategy could be cost effective, it might justify conducting a clinical trial to test the hypothesis that genetic test results would increase adherence to warfarin therapy, which may lead to a lower incidence of preventable strokes. In order to provide cost effectiveness estimates that could be used to justify or design such a clinical trial, we investigated whether the use of genetic information to increase adherence could be cost effective over a range of adherence rates.

## Methods

### Model structure and overview

**Fig. 1:**
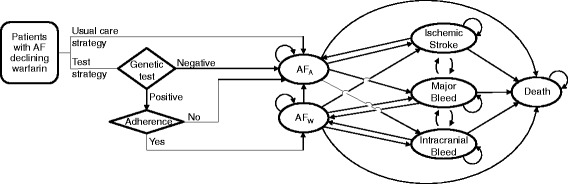
Schematic representation of patient care strategies and Markov model. Diamonds represent decision nodes, ovals represent Markov states. Arrows represent potential transitions between decision nodes and states. Abbreviations: AF_A_, atrial fibrillation patients on aspirin therapy; AF_W_, atrial fibrillation patients on warfarin therapy

Our analysis considered a hypothetical population of 1000 patients with AF who were prescribed warfarin to prevent future ischemic stroke events but who declined to adhere to warfarin therapy. We developed a Markov model to evaluate the payer-perspective cost-effectiveness of two patient care strategies (Fig. 1). One strategy is usual care: patients who refused warfarin therapy received aspirin therapy. The other strategy is a test strategy: patients were tested for genotypes of two variants in the 4q25 locus (rs2200733 and rs10033464). A fraction of patients with positive test results (having at least 1 risk allele of either variant) were assumed to be motivated to adhere to warfarin therapy. The remaining patients in the test strategy received aspirin therapy. The model compared expected costs and outcomes of these two strategies. Incremental cost-effectiveness ratios (ICERs) were calculated as the difference in costs between the usual care strategy and the test strategy divided by the difference in outcomes (expressed in quality adjusted life years, (QALYs) between the two strategies.

The model (Fig. 1) estimates the number of fatal and non-fatal stroke events, fatal and non-fatal major bleeding events, costs for drug and hospitalization, as well as QALYs in each strategy over a 5-year horizon using a 1-year cycle time. We assumed that warfarin reduced the annual rate of stroke compared with aspirin [21], that the rate of major bleeding events was lower among aspirin users compared with warfarin users [21], and that 40 % of the patients will have a positive genetic test result, based on the reported allele frequencies for these genetic variants [20].

The incidence of stroke and major bleeding events was calculated for each strategy over a 5 year time horizon using annual event rates for warfarin and aspirin as appropriate. We assumed that warfarin therapy will be discontinued for those patients who would experience an intracranial bleeding or other major bleeding event and that those patients on aspirin therapy would convert to warfarin therapy after an ischemic stroke event. For both strategies, we assumed that patients who initially declined warfarin therapy would convert to warfarin at an annual rate of 2.5 %. This annual rate of conversion to warfarin would be 2-fold more likely for test-positive patients who initially declined warfarin and 2-fold less likely for test-negative patients. One-time and subsequent monthly costs of stroke and major bleeding events were based on published 2005 estimates. A 3 % annual discount rate was applied to costs and utilities. Patient utility estimates for stroke, intracranial bleeding, and other major bleeding events were based on published estimates. Patients with fatal events were assigned a utility weight of 0. A summary of base case event rates, utilities, and costs as well as the respective sources for these values is provided in Table 1.

**Table 1:** Model Variables: base case patient probabilities, costs, and utility estimates

Variable	Base-Case	Range	Reference
Probabilities			
Baseline rate of stroke on aspirin, %/year	4.5	3–6	[23, 24]
Relative risk of stroke with warfarin compared with aspirin	0.48	0.37–0.63	[23, 24]
Baseline rate of major hemorrhage (including ICH) on warfarin, %/year	2.5	2–4	[23, 24]
Relative risk of hemorrhage with aspirin compared with warfarin	0.59	0.50–0.70	[23]
Fraction of ICH among major hemorrhage events	0.2	NA	[22]
Fraction of fatal strokes among stroke events on warfarin	0.082	NA	[24]
Fraction of fatal strokes among stroke events on aspirin	0.179	NA	[24]
Fraction of fatal events among ICH	0.364	NA	[25]
Fraction of fatal major hemorrhages (excluding ICH)	0.049	NA	[25]
Costs ($)			
Warfarin annual cost	180	±50 %	[24]
Aspirin annual cost	10	±50 %	[24]
Fatal ischemic stroke	12130	±50 %	Assumption, based on [24]
Average one-time cost of non-fatal stroke on warfarin	9667	±50 %	Calculated based on [24]
Average monthly cost of non-fatal stroke on warfarin	2652	±50 %	Calculated based on [24]
Average one-time cost of non-fatal stroke on aspirin	9610	±50 %	Calculated based on [24]
Average monthly cost of non-fatal stroke on aspirin	2168	±50 %	Calculated based on [24]
Intracoronary hemorrhage (ICH) one-time cost	31810	±50 %	[24, 26, 27]
Intracoronary hemorrhage (ICH) monthly costs	4690	±50 %	[24, 26, 27]
Major hemorrhage (excluding ICH)	3620	±50 %	[24, 26, 27]
Utilities			
Healthy on warfarin	0.987	unchanged	[28, 29]
Healthy on aspirin	0.998	unchanged	[28, 29]
Non-fatal stroke on warfarin, weighted average	0.476	±20 %	[24]
Non-fatal stroke on aspirin, weighted average	0.426	±20 %	[24]
Non-fatal ICH	0.4	±20 %	Assumption
Recurrent stroke	0.12	±20 %	[29]

### Sensitivity analyses

The adherence rates to warfarin among those with a positive 4q25 test result were varied from 1 to 50 % using base-case values. Sensitivity analyses were performed to examine the effect of varying key parameter estimates. One-way sensitivity analysis included the following parameters: the annual rate of stroke among aspirin users (varied between 3 and 6 %), the risk reduction of stroke by warfarin compared with aspirin (0.37 to 0.63), the annual rate of major bleeds among warfarin users (2 to 4 %), the relative risk of major bleeds by aspirin compared with warfarin (0.5 to 0.7), the cost of genetic testing ($50 to $200), the costs associated with events (strokes, major bleeds) and drugs (varied simultaneously 50 % above and below the base-case values), and utility estimates of the non-fatal states in the model (varied simultaneously 20 % above and below the base-case values). A probabilistic sensitivity analysis (PSA) was performed using a Monte Carlo simulation of 10,000 trials by drawing the values of the baseline parameters from triangular distributions. The contribution of baseline parameters to the variation observed in PSA was evaluated from the correlation between the variance of each parameter in the PSA model and the variance of the PSA outcome.

## Results

### Base-case analysis

**Fig. 2:**
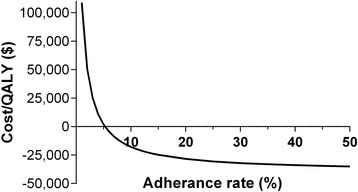
Adherence to warfarin therapy among test-positive patients and cost/QALY. The cost-effectiveness of genetic testing (ICER in US dollars) as a function of the initial rate of adherance to warfarin among test positive patients who initially decline warfarin therapy

We estimated the costs and utilities over a 5 year horizon of two health-care strategies among 1000 patients with AF who were prescribed warfarin to prevent future ischemic stroke events but who had declined warfarin therapy. The first strategy was usual care (aspirin therapy and no genetic test). The second strategy (test strategy) used genetic testing for risk of AF to motivate patients to adhere to the prescribed warfarin therapy (Fig. 1). We modeled testing of two genetic variants associated with risk of AF in the 4q25 locus (rs2200733 and rs10033464). We assumed that those who carry at least 1 risk allele of either variant (test positive) would be motivated to adhere to warfarin therapy. We first investigated the fraction of the test positive patients that would need to adhere to warfarin therapy in order to make the test strategy cost effective. We found that under the base-case assumptions (Table 1), the test strategy would be cost-effective compared with the usual care (cost/QALY = $47,148) if 2.1 % or more of the test positive patients choose to adhere to warfarin therapy. The test strategy would become a cost saving strategy if more than 5.3 % of the test positive patients adhere to warfarin therapy (Fig. 2). And, if 20 % of the test positive patients adhere to warfarin therapy, 7 stroke events would be prevented and 3 extra-cranial major bleeding events would be caused for every 1000 patients tested. That is, the test strategy would dominate the usual care strategy: the test strategy would result in better outcomes (a net gain of 9 QALYs) and lower costs (a net saving of ~ $250,000) over a 5 year horizon compared with the usual care strategy (Table 2).

**Table 2:** Cost savings and utility gains of the test strategy compared with the usual care strategy for different adherence assumptions for 1000 patients

Adherence rate, %	Cost saving (loss), $	QALYs gained
1	(72,332)	0.7
2	(55,331)	1.1
3	(38,330)	1.5
4	(21,329)	2.0
5	(4,328)	2.4
7.5	38,175	3.5
10	80,678	4.5
15	165,683	6.7
20	250,689	8.8
25	335,694	11.0
30	420,699	13.1
40	590,710	17.5
50	760,721	21.8

### Sensitivity analyses

**Fig. 3:**
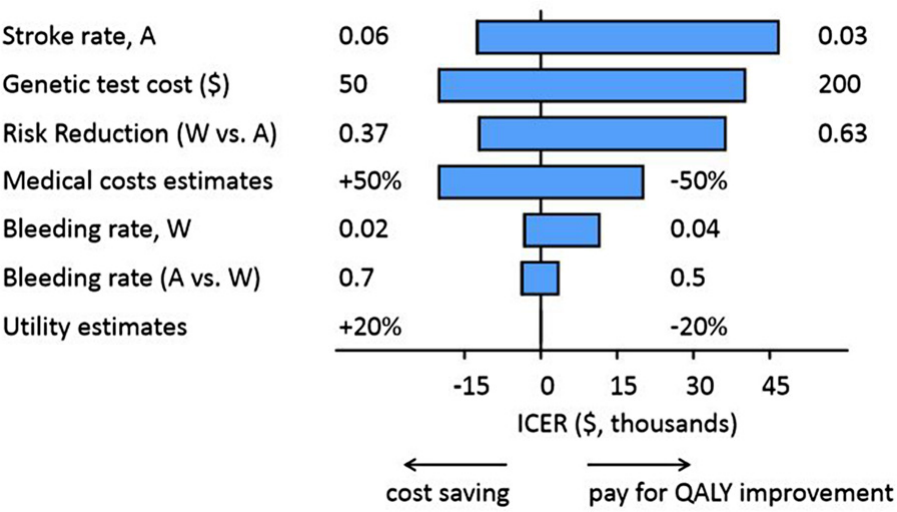
One-way sensitivity analysis. The effect of varying baseline parameters over clinically and economically relevant ranges on ICER. Parameter values used are indicated to the left and to the right of each blue bar. Abbreviation used: A, asprin; W, warfarin

**Fig. 4:**
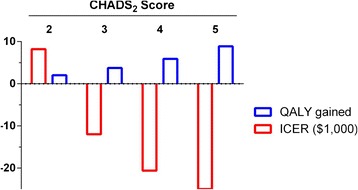
QALYs gained (per 1000 patients) and ICERs in patients with different CHADS_2_ scores

We examined the effect of the uncertainty in the input parameters on the model outcome by varying individual parameters from their baseline values. We used a 5.25 % adherence rate in the model to investigate the effect of baseline parameters when adherence rate would be cost neutral. We found that changing the input parameters had little effect on the cost-effectiveness of the test strategy compared with the usual care. When the parameters were within the ranges listed in Table 1, the test strategy remained cost effective (ICER < $47,000, Fig. 3). Moreover, varying the medical cost estimates, the bleeding rate among warfarin users, the relative bleeding rate among aspirin users vs. warfarin users, and the utility estimates all resulted in ICER estimates lower than $20,000 at their most unfavorable limit. Using the low end for the annual rate of stroke among aspirin users (3 %, compared with the baseline value of 4.5 %) resulted in an ICER of ~ $40,000, which was the largest ICER range for any individual baseline parameter. Since the rate of stroke events differs among individuals according to their CHADS_2_ score [24], we investigated QALY gained and ICERs in a model for patients with different CHADS_2_ scores (Fig. 4). When the test strategy was compared with the usual care strategy among patients with a CHADS_2_ score of 3 and greater, the test strategy would be dominating the usual care strategy: it would be cost saving and result in progressively more QALYs gained compared with the usual care as CHADS_2_ score increased.

**Fig. 5:**
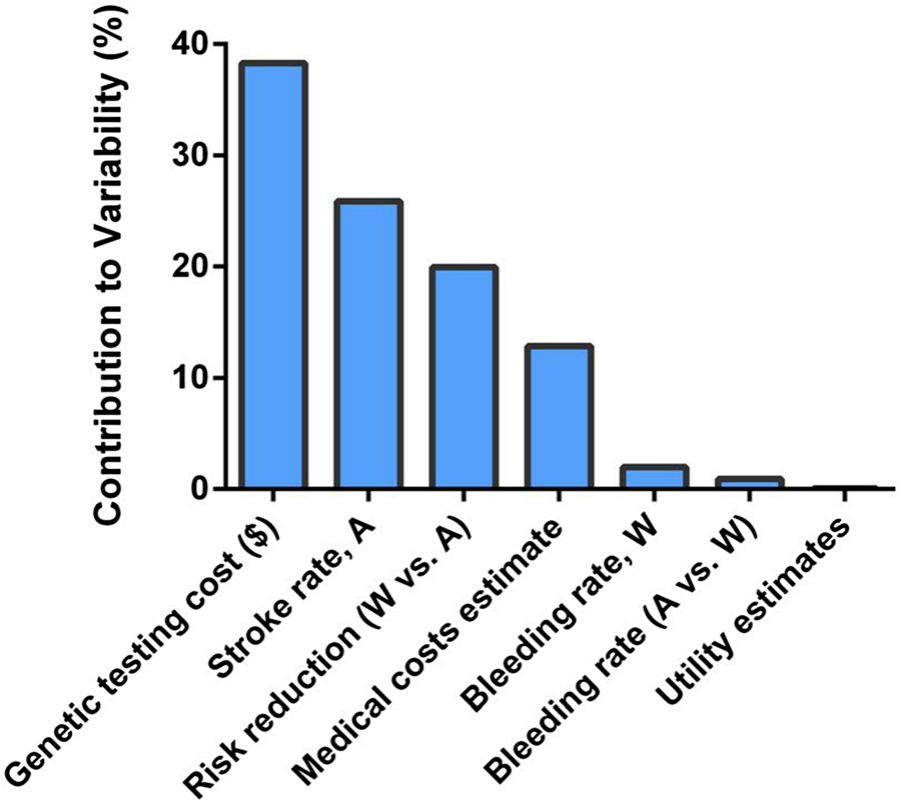
Probabilistic sensitivity analysis for the test strategy compared with the usual care: contribution of baseline parameter variability to overall model variability

**Fig. 6:**
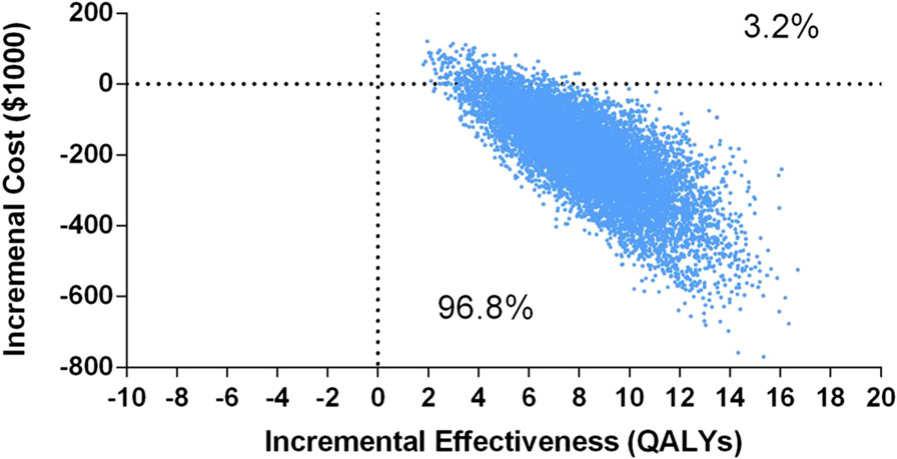
Probabilistic sensitivity analysis for the test strategy compared with the usual care strategy assuming 20 % of the test positive patients adhered to warfarin therapy. The cost-effectiveness plane shows the effect of simultaneously varying all model parameters on incremental cost (per 1000 patients, vertical axis) and incremental effectiveness (per 1000 patients, horizontal axis) in 10,000 Monte Carlo simulations

Using a probabilistic sensitivity analysis we investigated the contribution of each of these parameters to the overall variability of the ICER estimates. We found that 3 input parameters (the cost of genetic testing, annual stroke rate among AF patients who use aspirin, and the risk reduction of warfarin compared with aspirin) together account for more than 80 % of the variability in the model (Fig. 5). Assuming 20 % of the test positive patients adhered to warfarin therapy the test strategy was almost always (96.8 %) associated with lower cost and more QALYs than the usual care strategy (Fig. 6).

## Discussion

We compared the usual care strategy with a strategy that used genetic testing to motivate AF patients who declined physician prescribed warfarin therapy to reconsider and initiate therapy. We found that the test strategy could be cost effective even if only 2.1 % of test positive individuals adhered to warfarin therapy over a 5 year horizon. If less than 2 % of test positive individuals would adhere to warfarin therapy, this strategy becomes prohibitively expensive, costing more than $100,000 per QALY if adherence were to fall below 1 %. However, if adherence were to be greater than 5.3 %, this genetic test strategy would be cost-saving under the base-case assumptions.

A 5.3 % increase in adherence in response to genetic test information is smaller than what has been reported by in real-world studies. For example, among patients who were identified as having hypercholesterolemia based on their blood cholesterol levels, adherence to statin medication went up from 39 to 93 % following a genetic confirmation of their diagnosis [18]. Similarly, among all-comers with a newly-prescribed statin therapy, adherence increased from 45 to 63 % following genetic testing [19]. Thus, it seems that genetic testing results have the potential to increases in adherence of greater magnitude than that in the model we present. The specific effect of genetic testing on adherence to anticoagulation among patients with AF after genetic testing would need to be determined in a clinical study.

The model baseline parameters were based on published information. The effect of these parameters on the ICERs predicted by the model was modest. The low end of annual rate of stroke events among AF patients who use aspirin (but decline warfarin) resulted in the biggest ICER using baseline parameters. That is, if annual stroke rate among AF patients on aspirin was 3 %, the ICER would be ~ $47,000, however if the annual rate of stroke was at the high end (6 %) the genetic testing strategy would be dominating (cost-saving with better outcomes). Interestingly, if the annual rate of stroke among aspirin users were to be as low as 2 %, the number of strokes prevented by warfarin treatment would be offset by an equal number of major bleeds caused by warfarin treatment (1.5) regardless of treatment strategy. Conversely, a higher rate of strokes in subgroups of patients, which can be estimated by risk scores such as CHADS_2_, would make the test strategy more cost saving and gain more QALYs compared with the usual care strategy.

This study has several potential limitations. First, this study is based on a theoretical patient cohort and therefore used baseline parameters that were established in other real-life cohorts. Deviation from these baseline parameter estimates could affect the results. However, our sensitivity analyses established that variations in these parameters have only a small effect on the conclusions of this study. We found that the cost of the genetic test had the greatest influence on the range of total costs. We believe that the range of costs investigated ($50 to $200) was reasonable, because the genetic test considered in this study involved genotyping two single nucleotide polymorphisms (SNPs). And Medicare reimbursement for two SNPs was ~ $100 in 2013—this is based on the combined reimbursement for the Factor V Leiden SNP and for the Factor II (20210G > A) SNP [31]. We have based all other medical costs on published 2005 estimates [25]. However, inflating these costs to 2014 would result in more favorable outcome for the test strategy since the test strategy results in fewer events than the usual care strategy for any adherence rate. Second, we focused our analysis on adherence to warfarin as an oral anticoagulant. We believe an investigation of adherence to warfarin is important because warfarin remains first line therapy (recommendation 1A) in the 2014 AHA/ACC guidelines for the management of patients with atrial fibrillation [30]. New oral anticoagulants (NOACs: dabigatran, rivaroxaban, and apixaban) which are now available as anticoagulant option for some AF patients received a 1B recommendation. Patient adherence to NOACs is not likely better than to warfarin [31], presumably because bleeding event rates for warfarin are largely not different than that for most NOACs [32]. Moreover, the lack of approved antidote to NOACs can deter some patients from adhering to therapy. Third, we assumed the frequency of individuals who are carriers of a 4q25 risk allele (test positive) to be 40 %. This assumption is based on the reported allele frequencies of rs2200733 and rs10033464 in populations of European ancestry. The fraction of test positive individuals could vary in populations with a different ethnic ancestry. For example, among Yoruban in Ibadan, Nigeria, the fraction of test positive patients would be 86 % [33]. We elected to use the test positive information for European ancestry patients because most of the genetic association studies supporting the association of these SNPs with risk of AF were conducted in these populations. Our model could be easily adopted for other expected test positive fractions. Given these limitations, the results of this study should be cautiously considered when trying to extrapolate to real-life studies.

## Conclusions

In conclusion, we have demonstrated that under a wide range of input parameters, genetic testing to motivate adherence to warfarin therapy among AF patients eligible for this therapy would be cost effective. The range of cost effective adherence rates identified in this model could be used to aid in design of a clinical study that would test whether providing patients with information about their genetic AF risk would improve their adherence to warfarin therapy.
